# A novel device for detecting anaerobic threshold using sweat lactate during exercise

**DOI:** 10.1038/s41598-021-84381-9

**Published:** 2021-03-02

**Authors:** Yuta Seki, Daisuke Nakashima, Yasuyuki Shiraishi, Toshinobu Ryuzaki, Hidehiko Ikura, Kotaro Miura, Masato Suzuki, Takatomo Watanabe, Takeo Nagura, Morio Matsumato, Masaya Nakamura, Kazuki Sato, Keiichi Fukuda, Yoshinori Katsumata

**Affiliations:** 1 Department of Cardiology, Keio University School of Medicine, 35 Shinanomachi Shinjuku-ku, Tokyo, 160-8582 Japan; 2 Institute for Integrated Sports Medicine, Keio University School of Medicine, Tokyo, Japan; 3 Department of Orthopaedic Surgery, Keio University School of Medicine, 35 Shinanomachi Shinjuku-ku, Tokyo, 160-8582 Japan; 4 Department of Clinical Laboratory, Gifu University Hospital, Gifu, Japan; 5 Department of Clinical Biomechanics, Keio University School of Medicine, Tokyo, Japan

**Keywords:** Preventive medicine, Rehabilitation

## Abstract

The lactate threshold (LT1), which is defined as the first rise in lactate concentration during incremental exercise, has not been non-invasively and conveniently determined in a clinical setting. We aimed to visualize changes in lactate concentration in sweat during exercise using our wearable lactate sensor and investigate the relationship between the lactate threshold (LT1) and ventilatory threshold (VT1). Twenty-three healthy subjects and 42 patients with cardiovascular diseases (CVDs) were enrolled. During exercise, the dynamic changes in lactate values in sweat were visualized in real-time with a sharp continuous increase up to volitional exhaustion and a gradual decrease during the recovery period. The LT1 in sweat was well correlated with the LT1 in blood and the VT1 (r = 0.92 and 0.71, respectively). In addition, the Bland–Altman plot described no bias between the mean values (mean differences: − 4.5 and 2.5 W, respectively). Continuous monitoring of lactate concentrations during exercise can provide additional information for detecting the VT1.

## Introduction

Adequate regular physical activity is paramount to maintaining good health^1,2^ and preventing cardiovascular diseases (CVD). Current clinical practice guidelines and expert statements recommend aerobic exercise for patients with CVD^3–5^. Although an exercise test with respiratory gas analysis is the only non-invasive way to determine the ventilatory threshold (VT1)^6^ in clinical practice, VT1 assessment requires an expensive analyzer and expertise^5,7^. Additionally, it is incidentally difficult to confirm the VT1 because of oscillations in minute ventilation and inconsistencies among several factors such as the VE/VO_2_, the terminal exhaled O_2_ concentration, and the VCO_2_/VO_2_ slope^8^. Further, careful attention is necessitated when using a respiratory gas analyzer due to possible cross-infection. An alternative method is needed to detect VT1 easily and precisely without the need for a respiratory gas analyzer.

Flexible wearable sensing devices can yield important information about the underlying physiology of a human subject in a continuous, real-time, and non-invasive manner^9,10^. Sampling human sweat, which is rich in physiological information such as the sweat rate or sodium concentration, could enable non-invasive monitoring^11^. To date, sweat-based and non-invasive biosensors of lactate have been reported in research settings^12–14^ and have shown that sweat lactate is elevated in conjunction with exercise intensity. Therefore, the application of continuous lactate monitoring systems using wearable lactate sensors could influence exercise therapy in patients with CVD in clinical practice. However, these devices have not yet been applied in clinical practice, which might be due to unsuccessful miniaturization of devices and an operation that is easy to use, the inappropriate degree of accuracy as a medical device, or high cost. We have developed an innovative device in which sweat lactate may be monitored in a continuous, convenient, and non-invasive manner. We hypothesized that this device could be applied in clinical practice and successfully detected the lactate threshold (LT1) which is the first rise of lactate concentration during incremental exercise.

We aimed to investigate whether a usable device in the clinical setting would enable the continuous monitoring of sweat lactate during incremental exercise. Moreover, we elucidated the relationship among the lactate threshold in sweat (sLT), blood (bLT), and VT1 in healthy subjects and patients with CVD.

## Results

### In-vitro characterization of the lactate biosensor

**Figure 1:**
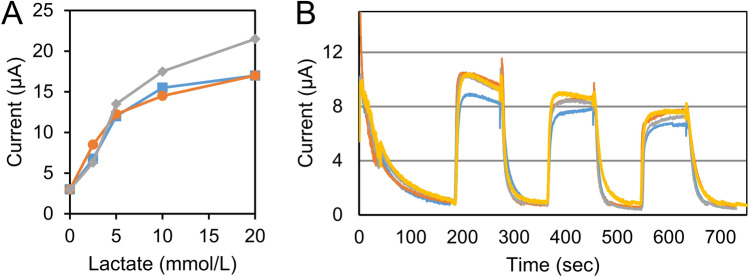
In-vitro characteristics of the sweat lactate sensor chip. (**A**) Amperometric response to increasing lactate concentration from 0 to 20 mM (0, 2.5, 5, 10, and 20 mM) in phosphate buffer (pH 7.0); the graph shows the corresponding calibration plots of the sensor. Applied voltage = 0.16 V versus Ag/AgCl. The data were obtained from three samples. (**B**) Reproducibility and long-term stability of the sweat lactate sensor; the graph shows amperometric response to l-lactic acid solution adjusted to 10 mM repeatedly three times for 90 s. Data recording was paused for 90 s for each response. The data were obtained from four samples.

Figure 1 shows the amperometric response of the lactate biosensor to increasing lactate concentrations in the physiological range of 0–10 mmol/L. The biosensor responded linearly to the lactate concentrations, especially in the ranges from 0 to 5 mmol/L, with a sensitivity of 2.4 A/mM (Fig. 1A). The range of 0–5 mmol/L was important in determining the LT1 because lactate concentrations around 2 mmol/L are related to LT1 / VT1^6^. Further, the sensors responded quickly and with almost the same value to L-lactate acid three times repeatedly (Fig. 1B).

### Study subjects

The baseline characteristics of the healthy subjects are summarized in Table 1. The healthy subjects were predominantly male (91%), with a median age of 20 (IQR 20–21) years. Tables 1 and 2 demonstrates the patient background of patients with CVD. The patients were predominantly male (76%), with a median age of 63 years (interquartile [IQR], 54–71) and left ventricular ejection fraction (LVEF) of 50% (IQR, 31.7–58.5). Thirty-four (83%) patients were taking beta-blockers.

**Table 1:** Baseline characteristics of healthy subjects and patients.

Demographic and anthropometric data	Healthy subjects (n = 23)	Patients (n = 42)
Age, years (median, IQR)	20 (20, 21)	63 (54, 71)
Male, n (%)	21 (91)	32 (76.2)
Height, cm (median, IQR)	171 (165, 175)	165 (159, 172)
Body weight, kg (median, IQR)	62 (58, 68)	62 (57, 71)
BMI, kg/m^2^ (median, IQR)	22 (20, 23)	23 (21, 25)
Hypertension, n (%)	–	12 (28.6)
Diabetes, n (%)	–	9 (21.4)
Dyslipidemia, n (%)	–	22 (52.4)
NYHA ≧3	–	19 (45.2)
Device, n (%)	–	4 (9.5)
**Laboratory data**		
Hemoglobin, g/dL (median, IQR)	–	13.7 (12.5, 14.6)
Creatinine, mg/dL (median, IQR)	–	0.9 (0.8, 1.1)
BNP, pg/mL (median, IQR)	–	146.8 (35.6, 328.0)
**Echocardiography data**		
LVEF, % (median, IQR)	–	49.5 (31.7, 58.5)
**Medications**		
Beta-blocker, n (%)	–	34 (82.9)
ACEI or ARB, n (%)	–	24 (58.5)
Statin, n (%)	–	19 (45.2)
Antiplatelet drug, n (%)	–	16 (38.1)
Anti-arrhythmic drug, n (%)	–	3 (7.1)
**Cardiopulmonary test data**		
VO_2_ at VT1, ml/kg/min (median, IQR)	–	10.5 (9.7, 12.3)
VT1, sec (median, IQR)	–	429.0 (391.5, 473.2)
Peak VO_2_, mL/kg/min (median, IQR)	–	15.9 (12.4, 18.9)
%Peak VO_2_, % (median, IQR)	–	67.5 (53.7, 79.7)
VE/VCO_2_ slope (median, IQR)	–	32.2 (28.6, 38.1)

*ACEI* angiotensin-converting enzyme inhibitor, *ARB* angiotensin receptor blocker, *BMI* body mass index, *BNP* B-type natriuretic peptide, *IQR* interquartile range, *LVEF* left ventricular ejection fraction, *NYHA* New York Heart Association Functional Classification, *VE/VCO*_*2*_ ventilation-carbon dioxide production, *VO*_*2*_ oxygen uptake, *VT1* ventilatory threshold.

**Table 2:** Respiratory gas data during exercise in the patients.

	Rest	Warm-up	VT1	Peak
HR, bpm	70 (62, 82)	79 (70, 92)	96 (85, 109)	127 (113, 137)
SBP, mmHg	107 (91, 123)	118 (99, 130)	127 (110, 140)	142 (116, 166)
DBP, mmHg	69 (62, 80)	76 (67, 86)	75 (65, 82)	79 (72, 90)
VO_2_, mL/kg/min	3.6 (3.4, 4.1)	6.5 (5.6, 7.1)	11.5 (9.7, 12.3)	15.9 (12.4, 18.9)
RQ	–	–	0.89 (0.86, 0.97)	1.15 (1.08, 1.20)
WR (W)	–	0	46 (37, 58)	77 (62, 107)
VE/VCO_2_ slope	32.3 (28.6, 38.1)			

All values are presented as medians and IQRs.

*DBP* diastolic blood pressure, *HR* heart rate, *IQR* interquartile range, *RQ* respiratory quotient, *SBP* systolic blood pressure, *VE/VCO*_*2*_ ventilation-carbon dioxide production, *VO*_*2*_ oxygen uptake, *VT1* ventilatory threshold, *WR* work rate.

### Monitoring of the lactate in sweat during exercise

**Figure 2:**
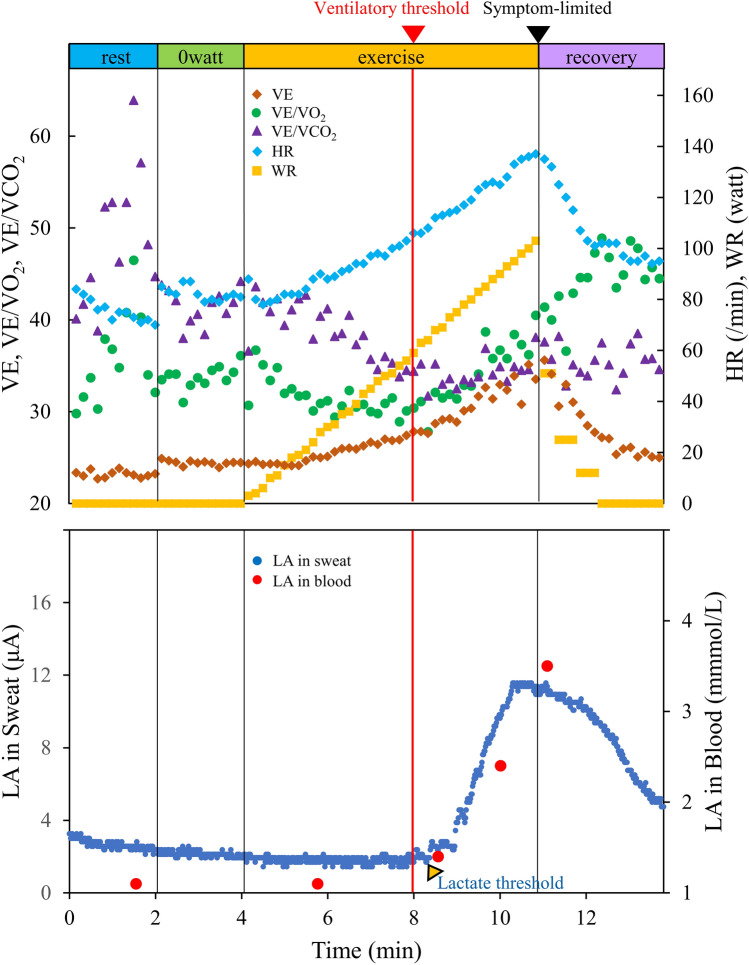
Imaging of the lactate in the sweat during incremental exercise. Representative graphs (dots) of the lactate in sweat (LA in sweat; dark blue) and lactate in blood (LA in blood; red) during exercise with a RAMP (15 W/min) protocol ergometer are shown in the lower panel. The respiratory gas data was shown in the upper panel. *HR* heart rate, *LA* lactate, *VE* ventilatory equivalent, *VE/VCO*_*2*_ ventilation-carbon dioxide production, *VE/VO*_*2*_ ventilation-oxygen uptake, *WR* work rate.

Figure 2 and Online Supplemental Video S1 show the lactate values in sweat during incremental exercise. Dynamic changes in sweat lactate values during the exercise tests were continuously measured and projected on the wearable device without delay in both the healthy subjects and a subset of patients with CVD. At the commencement of the cycling activity, negligible current response was measured by the lactate biosensor due to the lack of sweat. At the onset of sweating, lactate was released from the epidermis, and was selectively detected by the LOx-based biosensor. During the exercise, a drastic increase in sweat lactate values was observed as the cycling continued up to volitional exhaustion (Fig. 2). At the end of the exercise period, sweat lactate values continued to decrease relatively slowly, compared to the decrease in heart rate.

### Predictors associated with non-response in the lactate sensor

In patients with CVD, changes in sweat lactate values during exercise were similar to those in healthy subjects. However, 19 cases had steady low lactate values after starting the exercise until the recovery state (Online Fig. S1), suggesting a lack of sweat even with a maximum exercise load. Logistic regression analysis was performed to identify factors associated with non-response in the lactate sensor. The results of the univariate analyses are shown in Table 3. New York Heart Association functional classification (NYHA) 3 and low peak VO_2_ were associated with non-response in the lactate sensor among patients with CVD (odds ratio [OR], 0.06; 95% confidence interval [CI] 0.01–0.24; and OR, 1.18; 95% CI 1.02–1.42, respectively). Conversely, age, B-type natriuretic peptide, and LVEF were not associated with a non-response in the lactate sensor (OR 1.03 [95% CI 0.66–1.61], OR 0.95 [95% CI 0.84–1.05], and OR 1.17 [95% CI 0.95–1.48], respectively).

**Table 3:** Predictors of factors associated with response in the lactate sensor.

Factor	Odds ratio (95% CI)	P-value
Age, year (per 10-point increase)	1.03 (0.66–1.61)	0.883
Male	2.19 (0.52–10.08)	0.288
Height, cm (per 10-point increase)	1.68 (0.82–3.75)	0.173
Body weight, kg (per 10-point increase)	1.30 (0.78–2.34)	0.339
BMI, kg/m^2^	1.03 (0.88–1.21)	0.748
Hypertension	0.48 (0.12–1.84)	0.285
Diabetes	0.59 (0.13–2.62)	0.485
NYHA 3 (versus ≤ 2)	0.06 (0.01–0.24)	< 0.001
Hemoglobin, g/dL	1.18 (0.80–1.78)	0.414
Creatinine, mg/dL	0.93 (0.01–1.02)	0.736
BNP, pg/mL (per 50-point increase)	0.95 (0.84–1.05)	0.316
LVEF, % (per 5-point increase)	1.17 (0.95–1.48)	0.153
Beta-blocker	4.04 (0.75–31.20)	0.124
ACEI or ARB	1.24 (0.35–4.41)	0.732
VO_2_ at VT1, mL/kg/min	1.25 (0.95–1.71)	0.136
Peak VO_2_, mL/kg/min	1.18 (1.02–1.42)	0.044
VE/VCO_2_ slope (per 5-point increase)	0.88 (0.57–1.31)	0.519

*ACEI* angiotensin-converting enzyme inhibitor, *ARB* angiotensin receptor blocker, *BMI* body mass index, *BNP* B-type natriuretic peptide, *CI* confidence interval, *LVEF* left ventricular ejection fraction, *NYHA* New York Heart Association Functional Classification, *VE/VCO*_*2*_ ventilation-carbon dioxide production, *VO*_*2*_ oxygen uptake, *VT1* ventilatory threshold.

### Relationship among the sLT and bLT

**Figure 3:**
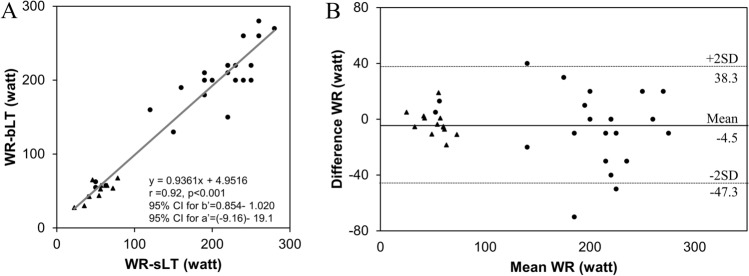
Validity testing of the WR at the sLT and bLT. (**A**) The graph shows the relationship between the work rate (WR) at the sLT and bLT. (**B**) The graph shows the Bland–Altman plots, which indicate the respective differences between WR at the sLT, and bLT (y-axis) for each individual against the mean of the WR at the sLT, and bLT (x-axis). Triangles indicate the data of patients and circles indicate the data of healthy subjects. *r* correlation coefficient, *95% CI for b’* 95% confidence interval for the slope, *95% CI for a’* 95% confidence interval for y-intercept, *SD* standard deviation.

The conversion from the steady low lactate values to the continuous increase easily detected in all healthy subjects and 23 patients with response in the lactate sensor (Fig. 2), was defined as sLT. Among the 23 CVD patients, the monitoring of blood lactate concentration during exercise was only enabled by 13 patients. Combining these 13 patients and all healthy subjects, the relationships between the WR-sLT and WR-bLT were investigated (Fig. 3A), which described a strong relationship between each threshold (r = 0.92, P < 0.001). The Bland–Altman plot revealed that the mean difference between each threshold was − 4.5 W, and that there was no bias between the mean values, which displayed strong agreements between the WR-sLT and WR-bLT (Fig. 3B). Least-product regression analysis indicated no evidence of a fixed bias and a proportional bias (95% CI for y-intercept, − 9.16 to 19.1; 95% CI for the slope 0.854–1.020).

### Relationship among the sLT and VT1

**Figure 4:**
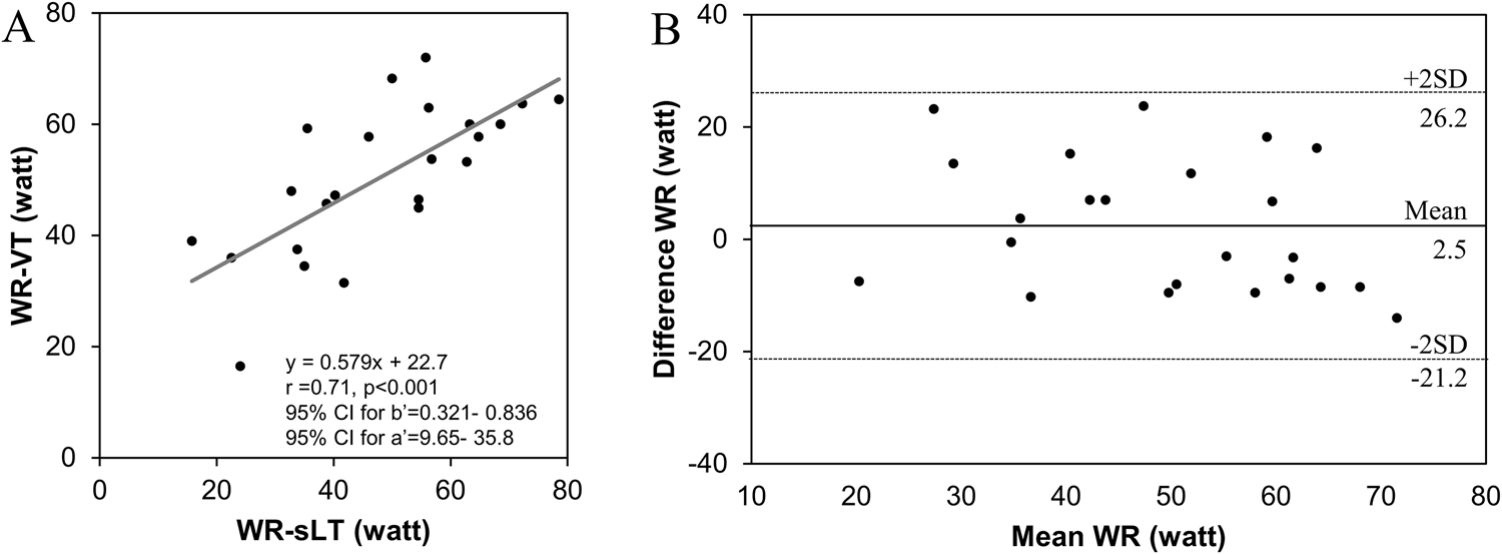
Validity testing of the WR at the sLT and bLT or VT1. (**A**) The graph shows the relationship between the work rate (WR) at the lactate threshold in sweat (WR-sLT) and WR-ventricular threshold (WR-VT1). (**B**) The graph shows the Bland–Altman plots, which indicate the respective differences between WR-sLT, and WR-VT1 (y-axis) for each individual against the mean of WR-sLT, and WR-VT1 (x-axis). *r* correlation coefficient, *95% CI for b’* 95% confidence interval for the slope, *95% CI for a’* 95% confidence interval for y-intercept, *SD* standard deviation.

Similarly, a good correlation was observed between the WR-sLT and WR-VT1 (r = 0.71, P < 0.001; Fig. 4A). The Bland–Altman plot described a strong agreement in the patients with CVD (Fig. 4B; the mean difference between each threshold, 2.5 W). Least-product regression analysis indicated a fixed bias (y-intercept, 22.7) and a proportional bias (slope, 0.57) between each threshold.

## Discussion

The most striking result to emerge from our findings is that the non-invasive lactate sensor enabled continuous and real-time measurement of sweat lactate values during an incremental exercise test. Furthermore, sLT strongly correlated with both bLT and VT1 in a subset of patients with CVD as well as in healthy subjects. The real-time lactate monitoring in sweat could be applicable for the detection of the LT1.

Lactate has three roles including, acting as a major energy source, a gluconeogenic substrate, a cell signaling molecule, and is used as optimal fuel for working muscles^15^. Muscle lactate production is essential to increase exercise performance^16^, and lactate should be measured during exercise to track an individual’s performance and exertion level^17,18^. Lactate values can be conventionally acquired via clinical labs or point-of-care devices^19,20^; unfortunately, such approaches do not support continuous, real-time measurements, a fact that limits their utility to applications where stationary, infrequent tests are sufficient. Conversely, our devices captured the sweat lactate value during exercise in a real-time, continuous, and non-invasive manner in a subset of patients with CVD in addition to healthy subjects. Sweat lactate has been affected by the production of lactate in the body and the rate of sweating and metabolic dynamics in sweat glands^17,21^. In addition, lactate is secreted into sweat, mirroring the intensity of exercise, but its concentration decreases with increasing sweat volume^17^. Therefore, the sweat lactate concentration has been reported not to have reflected the blood lactate concentration in specific circumstances, such as during vigorous exercise^22^. By combining the sweat lactate concentration with the sweat rate, the amount of lactate excreted from sweat may be calculated. Sweat lactate discharge may be more predictive of blood lactate levels than lactate concentrations. Further research is also warranted to examine this relationship.

Our sweat lactate sensor enabled the collection of sweat immediately after discharge unlike the other devices where sweat data represented a mixed state including previously discharged sweat. Therefore, the sensor used in this study successfully captured a rise in sweat lactate without delay. Moreover, despite the discrepancy between the sweat and blood lactate concentrations during vigorous exercise, there was no fixed and proportional biases between the WT-sLT and WT-sLT, which indicated that a rise in blood lactate coincided with that in sweat lactate during an incremental exercise. An increase in lactate production from muscle cells, reflecting the LT1, may induce a simultaneous rise in sweat lactate through a change in autonomic nervous balance, hormones, acid–base equilibrium, and metabolic dynamics^23–26^. However, the mean difference between WR-sLT and WR-VT1 was small, but the SD was rather large, and the presence of fixed and proportional bias was also indicated, which suggested a poor relationship between each threshold. This may have been caused by the difficulties in confirming the VT1 in some cases due to the inconsistencies among an increase in the ventilatory equivalent, excess CO_2_, and modified V-slope methods.

Flexibility is crucial for unobtrusive wearable devices that cause no hindrance or irritation to the wearer. Recent advances in fabrication techniques have enabled the design of wearable sensing devices in thin, conformal form that naturally comply with the smooth curvilinear geometry of human skin, thereby enabling close contact that is necessary for robust physiological measurements and monitoring of chemicals and electrolytes in sweat^27–29^. Our sensor was highly flexible and can be smoothly adjusted to curved surfaces using PET substrates. The upper arm and forehead have a high-sweat rate during physical excursion^30–32^ and can thus, serve as an appropriate area to measure lactate values in human sweat. Additionally, the epidermis and muscle tissues around the upper arm or forehead do not experience complex 3D strains and remain stable even during intense physical activities. Therefore, the sensor was attached to the upper arm in the healthy subjects considering easy operability for the use in outdoor sports or exercise. Conversely, in patients with CVD, who experience less sweating than healthy subjects, the sensor was attached to the forehead which has a higher-sweat rate^32^. However, it was not possible to continuously measure lactate in patients with NYHA3 or low peak oxygen uptake. Non-response in the sensor indicates a lack of sweat during exercise, which could be caused by intravascular dehydration by diuretics, abnormality of autonomic nervous balance, such as dominant sympathetic activity, or frailty due to heart failure. Further research is also warranted to develop wearable devices to monitor lactate values in patients without efficient sweat production.

In clinical practice, exercise testing with respiratory gas analysis is the most useful way to determine VT1. However, it is often difficult to determine VT1 because of oscillations in minute ventilation and inconsistencies among several factors such as the VE/VO_2_, the terminal exhaled O_2_ concentration, and VCO_2_/VO_2_ slope^8^. Furthermore, the use of a respiratory gas analyzer has a cross-infection possibility because of the closed circuit. The determination of sLT using only sweat-based monitoring could overcome these problems, and the device developed and used here would be suitable for use in a remote patient monitoring or remote rehabilitation setting during isolation measures, such as that taken during the COVID pandemic. Further, real-time assessments of sweat lactate values through a wireless data transfer system can offer a rigorous aerobic exercise based on the day-to-day physical conditions of patients with CVD as well as healthy subjects (Online Fig. S2). This innovative system could improve persistency of cardiac rehabilitation in outpatients and relocate their therapy from hospitals to other institutions, such as commercial fitness clubs or even patients’ homes.

Our findings should be interpreted with the following limitations. First, because of the observational study design, we could not deny the influence of selection bias and unmeasured confounders regarding the effect on response in the lactate sensors. Second, our study had a relatively small number of cases and included no control group in which the sensors without lactate oxidase were used for comparison. To validate that lactate and not other sweat constituents was measured, a control experiment in which an unmodified (LOx-free) amperometric biosensor should be undertaken under the same experimental conditions. Future randomized-controlled studies with different medical centers are required to overcome these limitations. Third, sweat rate was not measured during exercise because of a lack of a sweat rate sensor in our device. Therefore, it is unknown whether nonresponse in the lactate sensor is caused by a lack of sweat or rough contact necessary for robust physiological measurements. In addition, the exercise duration may be related to the amount of sweat. Exercise protocol improvements, such as a longer warm-up time, may overcome the lack of sweat in some cases. Further studies are needed to examine the relationship between nonresponse in the lactate sensor and a lack of sweat. Fourth, the sweat lactate sensor used in this study did not operate in an environment with a lack of sweat. It was thus not possible to measure changes in sweat lactate in the low-intensity range where there was no-sweating and in patients who did not sweat during exercise, such as with NYHA3 or low peak O_2_ uptake.

## Conclusions

This was the first study to show real-time monitoring of sweat lactate values during incremental exercise in patients with CVD as well as in healthy subjects. Given the difficult situation of deciding VT1, the monitoring of lactate values in sweat could be helpful for improving the detection of VT1.

## Methods

### Lactate measurement device

L-lactic acid and hydroxymethylferrocene were obtained from Tokyo Chemical Industry Co., Ltd. (Tokyo, Japan). Phosphate buffer solution (PBS) (0.1 mol/L, pH 7.0) was purchased from the Nacalai Tesque, Inc. (Kyoto, Japan). L-LOx (LCO-301) was purchased from the Toyobo Corp. (Osaka, Japan). Water for molecular biology (H20MB0501) was obtained from Merck KGaA (Darmstadt, Germany). Water-soluble photocurable photosensitive resin (BIOSURFINE-AWP) was obtained from Toyo Gosei Co., Ltd. (Tokyo, Japan). Methanol was obtained from FUJIFILM Wako Pure Chemical Corporation (Osaka, Japan). The original printing electrode chip (DEP-CHIP) was procured from Bio-Device Technology, Inc. (Ishikawa, Japan).

### Instrumentation

**Figure 5:**
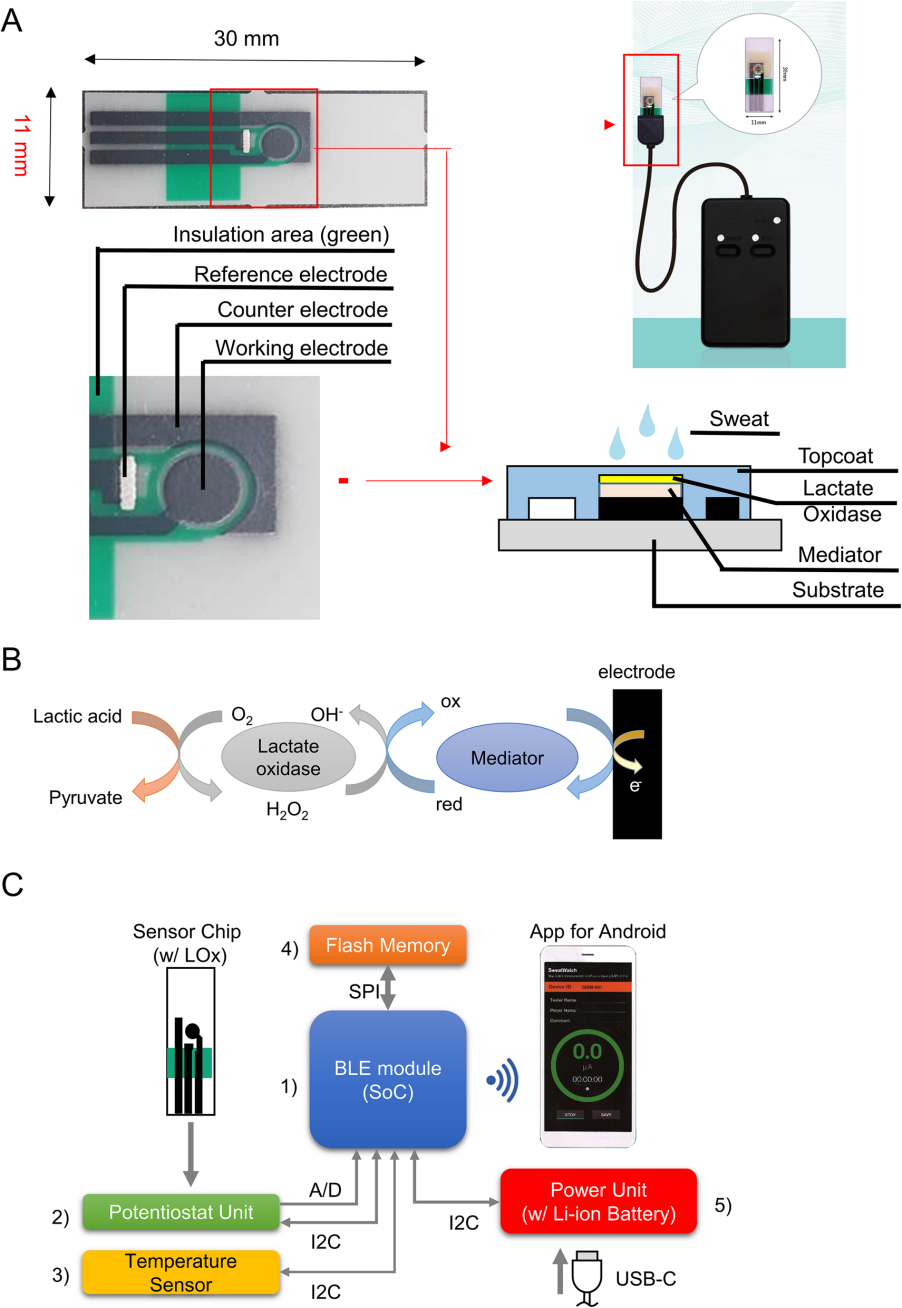
Fabrication and function of the sweat lactate sensor chip. (**A**) Parts composition of the lactate acid sensor chip. (**B**) Schematic diagram of the reagent layer and processes involved in the amperometric sensing of lactate acid on the working electrode. (**C**) The parts composition of device. (1) Bluetooth LE System-on-Chip: Taiyo Yuden EYSHCNZWZ, (2) Potentiostat unit: Texas Instruments DAC081C085 (DAC), Microchip Technology MCP6041T-I/OT and MCP6042T-I/MS (OP AMP), (3) temperature sensor: Ablic S-5851A, (4) flash memory: Winbond Electronics W25Q32JV(4 MB), (5) power unit: Texas Instruments BQ24232RGTR (Charger IC), Synergy ScienTech AHB512229PR (Li-ion Battery: 3.7 V, 295 mAh) and ON Semiconductor LC709203F (fuel gauge).

The original printed electrode chip (hereinafter referred to as "printed electrode"), which is the base of the lactate sensor chip, was designed using computer-aided design with a pattern shape consisting of three poles: an acting electrode, a counter electrode, and a reference electrode (Fig. 5A). Subsequently, PET substrates (Toray Industries, Inc., Tokyo, Japan) were fabricated using carbon ink, Ag/AgCl and insulating ink in a screen-printing process. These processes were outsourced to Bio-Device Technology, Inc (Ishikawa, Japan).

### Fabrication of lactate sensor chips

A total of 0.5 μL of hydroxymethylferrocene saturated methanol solution and 1.0 μL of L-LOX 0.5 wt% solution were applied to the working electrode of the printing electrode using a micropipette, and the working electrode was dried at room temperature (20–24 °C). The entire surface of the working electrode, counter electrode, and reference electrode was then coated with BIOSURFINE-AWP diluted to 3 wt% using pre-molecular biological water with an applicator to achieve a film thickness of 15 μm. Finally, the lactate sensor chip was fabricated by forming a protective film by exposure using a UV lamp (365 nm wavelength). The fabricated lactate sensor chips were kept refrigerated at 5 °C (Fig. 5A). When the biosensor contacts lactate, the immobilized Lox enzyme catalyzes the oxidation of lactate to generate pyruvate and H_2_O_2_. The Prussian blue transducer then selectively reduces the H_2_O_2_ to generate electrons to quantify the lactate concentration (Fig. 5B).

### Lactate sensor device

Lactate concentration was determined by the voltage of the working electrode (WE) on the sensor chip via a potentiostat unit, driven through the I2C interface. During measurement, elapsed time from start (in seconds), A/D converted voltage (equivalent to lactate concentration), and temperature (in Celsius) were stored as 10-byte binary strip data on flash memory using SPI. An in-house mobile application then received the data from a connected device at 1 s intervals for 10–20 min. The operating voltage was regulated to 3 V via an LDO regulator. The battery was charged via a USB Type-C cable and of the in-house mobile application was regularly notified its level. Power on/off and Bluetooth LE communication status had been indicated for the user with LEDs (Fig. 5C).

### In-vitro studies

The lactate concentration in human sweat depends on metabolism and level of exertion, and typically ranges from 0 to 20 mmol/L. A wide linear-detection range coupled with a fast response time is thus essential for continuous epidermal monitoring of lactate. Therefore, the electrochemical characterization of the LA sensor chip was performed using l-lactic acid solutions in 0 (pH 7.0), 2.5 (pH 7.0), 5 (pH 6.9), 10 (pH 6.8), and 20 (pH 6.6) mmol/L prepared in 0.1 mol/L phosphate buffer solution (PBS). Then, the three lactate sensor tips were evaluated using chronoamperometry at an overprinting voltage of 0.16 V (versus Ag/AgCl). In addition, the four sensor tips were evaluated for a total of three times with L-lactate solution adjusted to 10 mmol/L, to evaluate the long-term stability of the sensor. The electrochemical characterization was performed at room temperature (20–24 ℃using an electrochemical analyzer from Grace Imaging. Inc.

### Lactate measurement in humans

#### Study sample and ethical approval

Twenty-three healthy subjects were recruited, and 42 consecutive patients with CVD (e.g., heart failure, cardiomyopathy, or coronary artery disease) who underwent incremental exercise testing between November 2019 and November 2020 at Keio University Hospital were enrolled. The healthy subjects had a broad spectrum of aerobic capacities and fitness levels, but were not athletes, and had no comorbidities, such as hypertension, diabetes, or active lung diseases. Exclusion criteria for the patients with CVD included 2 or 3 degree-conduction block without a cardiac implantable electronic device, severe pulmonary hypertension, decompensated heart failure, more than severe primary valvular heart diseases, and an acute phase of the acute coronary syndrome. The study protocol was approved by the Institutional Review Board of Keio University School of Medicine [permission number; 2014023, 20180357], and was conducted in accordance with the Declaration of Helsinki. All subjects provided written informed consent.

### Experimental procedure

The exercise tests were performed with the RAMP protocol ergometer in both healthy subjects and patients with CVD, simultaneously monitoring the changes in sweat lactate with a wearable lactate sensor. In all healthy subjects, the sensor was attached to the upper arm, and lactates in blood were measured every 2 min. Conversely, in patients with CVD, the sensor was attached to the forehead which has a higher-sweat rate compared to the upper arm as they were less likely to sweat than healthy subjects. All the patients underwent an exercise test with respiratory gas analysis. Among the 42 patients, 17 patients refused the blood lactate test during exercise because of the invasive procedure. In only 25 patients who provided written informed consent, blood lactate concentrations were measured during the exercise test^26,33^.

### Exercise testing protocol

On the day of the exercise test, the subjects avoided heavy physical activity before the test. The subjects performed the test in the upright position on an electronically braked ergometer (STRENGTH ERGO 8, Mitsubishi Electric Engineering Company, Japan). Following a 2-min rest to stabilize the heart rate and respiratory condition, the subjects performed a 2-min warm-up pedaling at 50 W for healthy men and at 0 W for healthy women and patients, and then exercised with a progressive intensity until the subjects could no longer maintain the pedaling rate (volitional exhaustion). At 1-min intervals, the intensity was increased by 20 W increments for healthy subjects, and 10 or 15 W increments for CVD patients (RAMP protocol). The pedaling frequency was set at 60 rev/min. The incremental exercise testing time ranged from 10 to 20 min, depending on the exercise capacities of each subject or patient. Once the exercise tests were terminated the subjects were instructed to stop pedaling and to stay on the ergometer for 3 min^26,33^.

### Respiratory gas analysis and Ventilatory threshold

The additional method is available in the supplemental material S1. The expired gas flows were measured using a breath-by-breath automated system (AEROMONITOR, MINATO MedicalScience CO., LTD., Osaka, Japan). The respiratory gas exchange, including ventilation (VE), oxygen uptake (VO_2_), and carbon dioxide production (VCO_2_), was continuously monitored and measured using a 10-s average. VT1 was determined using the ventilatory equivalent, excess CO_2_, and modified V-slope methods^8^. Three exercise testing experts, agreed on the VT1, independently from those who determined the sLT. First, two of three experienced researchers independently and randomly evaluated the VT1 of each subject using the three methods. The researchers used all three methods to assess concurrent break point and to eliminate false breakpoint. Second, if the VO_2_ values determined by the independent researchers were within 3%, then the VO_2_ values for the two investigators were averaged. Third, if the VO_2_ values determined by the independent evaluators were not within 3% of one another, a third researcher then independently determined VO_2_. The third VO_2_ value was then compared to those obtained by the initial investigators. If the adjudicated VO_2_ value was within 3% of either of the initial investigators, then two VO_2_ values were averaged^26,33^.

### Lactate threshold in blood

The blood lactate values were obtained via auricular pricking and squeezing the ear lobe gently to obtain a capillary blood sample every 2 min during the exercise test. The samples were analyzed immediately for the whole blood lactate concentration (mmol/L) using a standard enzymatic method on a lactate analyzer (LACTATE PRO2, ARKRAY, Japan)^34^.

The bLT was determined through graphical plots^35^. A visual interpretation was independently made of each subject by two experienced researchers to locate the first rise from baseline. If the independent determinations of the stage at LT1 differed between the two researchers, a third researcher adjudicated the difference by independently determining LT1. The three researchers then jointly agreed on the LT1 point.

### Lactate threshold in sweat

The sLT was defined as the first significant increase in lactate in sweat above the baseline based on the graphical plots and Change Finder scores calculated by Change Finder algorithm (Online Fig. S3). Several candidate points (change points) of sLT were extracted by applying Change Finder algorithm^36^ to the time-series data of the lactate values in sweat in the range from the start to the end of exercise. Two-step learning with a Sequentially Discounting AR (SDAR) model was used to accurately distinguish between outliers and change points in the Change Finder algorithm. Three researchers, independently of the researchers who analyzed respiratory gas exchange, jointly agreed on the point of sLT.

### Statistical analyses

The results are represented as median with an interquartile range (IQR) for continuous variables and as percentages for categorical variables, as appropriate. A univariable logistic regression analysis was performed to estimate the adjusted odds ratios (ORs) and 95% confidence intervals (CIs) for non-response in the lactate sensor. The relationships among the work rate (WR) at the sLT, bLT, and VT1 were investigated using the Pearson's correlation coefficient test. Additionally, the Bland and Altman technique was applied to verify the similarities among the different methods^37^. This comparison was a graphical representation of the difference between the methods and the average of these methods. Further, ordinary least products regression analysis was used to evaluate the fixed and proportional biases between each threshold^38,39^. All probability values were 2-tailed with P values < 0.05 considered statistically significant. All statistical analyses were performed with R version 3.6.3 (R Core Team, 2020, R: A language and environment for statistical computing. R Foundation for Statistical Computing, Vienna, Austria).

## Supplementary Information

Supplementary Information 1.

Supplementary Video 1.

## Abbreviations

sLT: Lactate threshold in sweat
bLT: The lactate threshold in blood
VT1: Ventilatory threshold
CVD: Cardiovascular disease
WR: Work rate
NYHA: New York Heart Association Functional Classification
VE: Ventilation
VO_2_: Oxygen uptake
VCO_2_: Carbon dioxide production
