# Supplementary material for: A novel device for detecting anaerobic threshold using sweat lactate during exercise
Source: Sci Rep. 2021 Mar 2;11:4929. doi: 10.1038/s41598-021-84381-9 (PMC7925537; doi:10.1038/s41598-021-84381-9)
Supplement: Supplementary file 1 — Supplementary Information 1. [file 41598_2021_84381_MOESM1_ESM.docx]

**Supplementary Material**

**Title:** A novel device for detecting anaerobic threshold using sweat lactate during exercise

**Authors:** Yuta Seki MD, Daisuke Nakashima MD, PhD, Yasuyuki Shiraishi MD, PhD, Toshinobu Ryuzaki MD, Hidehiko Ikura, MD, Kotaro Miura, MD, Masayasu Fujioka MS, Masato Suzuki BS, Takatomo Watanabe MD, PhD, Takeo Nagura MD, PhD, Morio Matsumato MD, PhD, Masaya Nakamura MD, PhD, Kazuki Sato, MD, PhD, Keiichi Fukuda, MD, PhD, Yoshinori Katsumata, MD, PhD

**Supplementary Methods**

**Exercise testing protocol**

All subjects were examined under standard conditions. Exercise tests were performed within 1-3 hours after a meal and any caffeinated beverages were restricted 3 hours before exercise. On the day of the exercise test, the subjects avoided heavy physical activity before the test. An incremental cycle exercise was performed in a quiet room maintained at a constant temperature (22-24°C). The subjects performed the test in the upright position on an electronically braked ergometer (Strength Ergo 8^®^, Mitsubishi Electric Engineering Company, Japan). Following a 2-minute rest to stabilize the heart rate and respiratory condition, the subjects performed a 2-minute warm-up pedaling at 50 W for healthy men and at 0 W for healthy women and patients, and then exercised with a progressive intensity until the subjects could no longer maintain the pedaling rate (volitional exhaustion). At 1-minute intervals, the intensity was increased by 20-W increments for healthy subjects, and 10 or 15-W increments for patients. The incremental exercise testing time ranged from 10-20 minutes, depending on the exercise capacities of each subject. The pedaling frequency was set at 60 rev/min. Once the exercise tests were terminated the subjects were instructed to stop pedaling and to stay on the ergometer for 3 minutes (recovery phase). A 12-lead electrocardiogram continuously recorded the heart rate for patients. The blood pressure was measured every minute with an indirect automatic manometer for patients.

**Respiratory gas analysis and ventilatory threshold**

The expired gas flows were measured using a breath-by-breath automated system (Aeromonitor^®^, MINATO MedicalScience CO., LTD., Osaka, Japan). The respiratory gas exchange, including ventilation (VE), oxygen uptake (VO_2_), and carbon dioxide production (VCO_2_), was continuously monitored and measured using a 10-second average. This system was subjected to a three-way calibration process, involving a flow volume sensor, gas analyzer, and delay time calibration. The peak VO_2_ was calculated as the average oxygen consumption during the last 30 seconds of exercise. The ventilation/carbon dioxide (ventilator efficiency) slope (VE-VCO_2_ slope) was based on data from the onset of exercise to the respiratory compensation point, and was obtained via linear regression analysis of the data acquired throughout the entire period of exercise. The resting heart rate was defined as the average of the heart rates in the sitting position for 2 minutes before the exercise.

VT was determined using the ventilatory equivalent, excess carbon dioxide, and modified V-slope methods. First, two of three experienced researchers independently and randomly evaluated the VT of each subject using the three methods. The researchers used all three methods to assess concurrent break point and to eliminate false breakpoint. Second, if the VO_2_ values determined by the independent researchers were within 3%, then the VO_2_ values for the two investigators were averaged. Third, if the VO_2_ values determined by the independent evaluators were not within 3% of one another, a third researcher then independently determined VO_2_. The third VO_2_ value was then compared to those obtained by the initial investigators. If the adjudicated VO_2_ value was within 3% of either of the initial investigators, then two VO_2_ values were averaged.

**Supplementary figures**

**Online Figure 1: Imaging of lactate concentration in sweat during incremental exercise in the patients with non-response to the sensor**


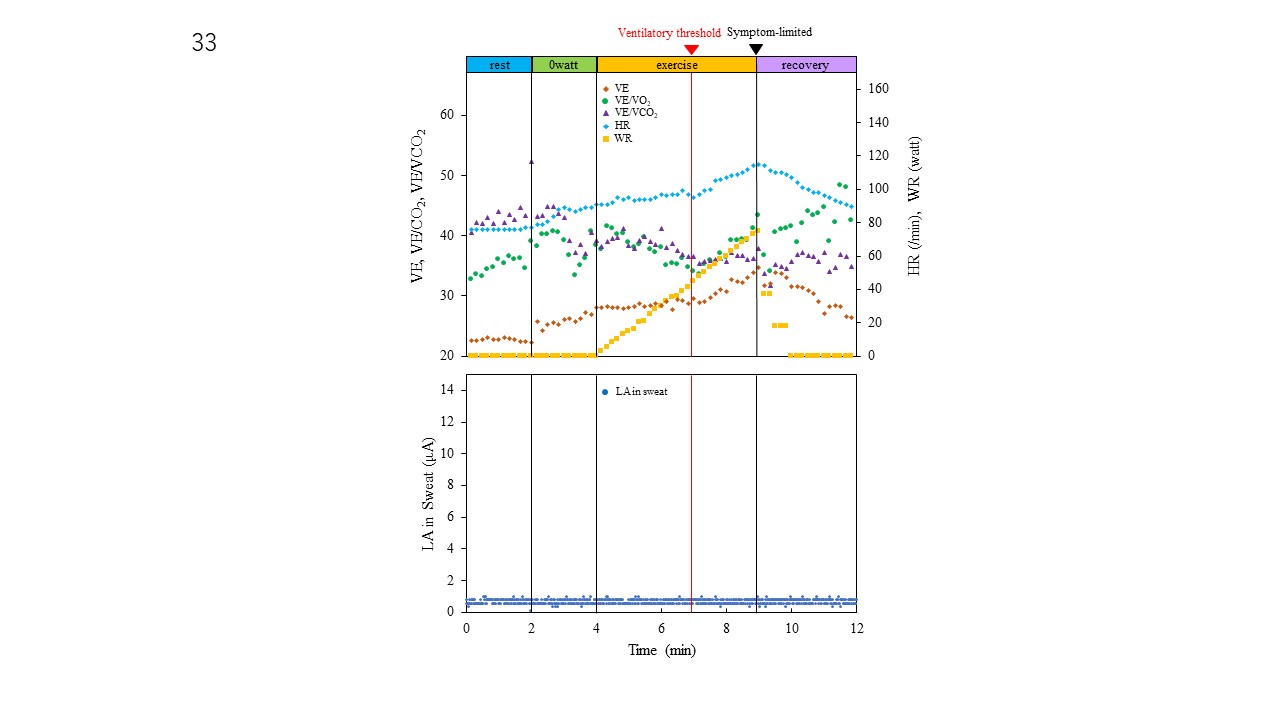


Representative graphs (dots) of the lactate acid in sweat (LA in sweat; dark blue) during exercise with a RAMP (15 W/min) protocol ergometer in patients with non-response to the sensor are shown in the lower panel. The respiratory gas data are shown in the upper panel. Abbreviation; HR = heart rate; LA = lactic acid; VE/VCO_2_ = ventilation–carbon dioxide production; VE/VO_2_ = indicates ventilation-oxygen uptake; WR = work rate.

**Online Figure 2. Schematic diagram of a wireless operation of the lactate-sensing device during fitness**


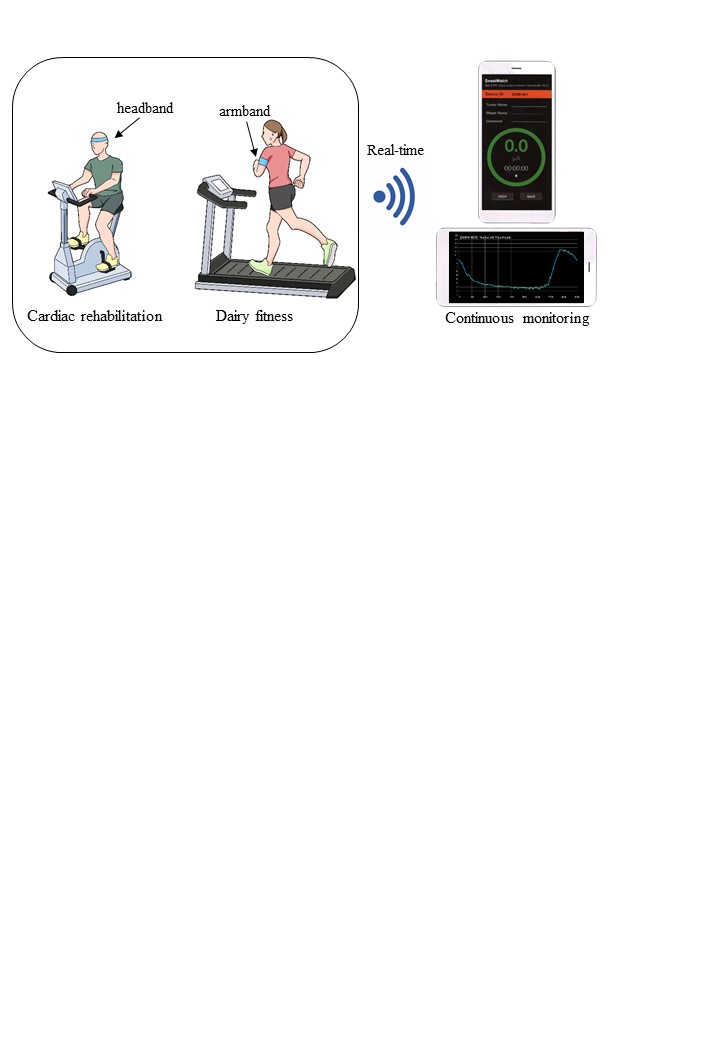


**Online Figure 3: Candidate points of the lactate threshold in sweat extracted using the Change Finder algorithm**

recovery

exercise

0 watt

rest

^
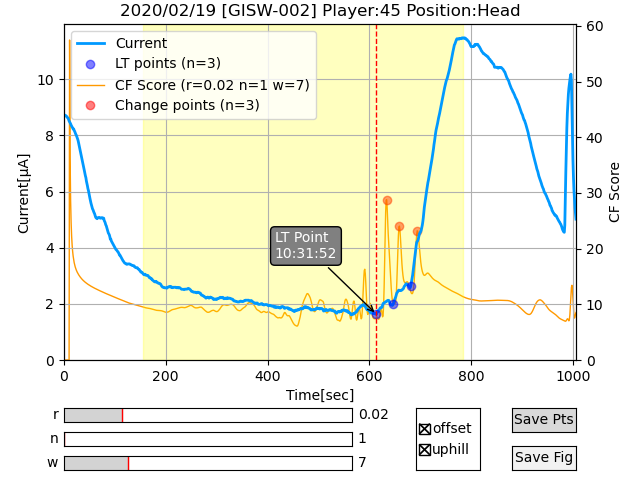
^

The data were obtained from the same patients as Figure 2. The orange line indicates the Change Finder (CF) score calculated by applying Change Finder algorithm to the time-series data of the LA values in sweat in the range from the start to the end of exercise (yellow area). Several candidate points of sLT (blue points) with high CF score were extracted.

**Video Legends**

**Online Video 1. Quantitative movie imaging of lactate concentration in sweat during incremental exercise**

A representative movie of the lactate acid concentration in sweat (blue line) during an exercise test with a RAMP (15 W/min) protocol ergometer are shown in the same patients as Figure 2.
